# Association between Myocardial Infarction and Periodontitis: A Meta-Analysis of Case-Control Studies

**DOI:** 10.3389/fphys.2016.00519

**Published:** 2016-11-04

**Authors:** Quan Shi, Bin Zhang, Na Huo, Chuan Cai, Hongchen Liu, Juan Xu

**Affiliations:** Institute of Stomatology, Chinese PLA General HospitalBeijing, China

**Keywords:** periodontitis, myocardial infarction, periodontal examination, risk factor, case-control study, meta-analysis

## Abstract

**Background and Objective:** Many clinical researches have been carried out to investigate the relationship between myocardial infarction (MI) and periodontitis. Despite most of them indicated that the periodontitis may be associated with an increased risk of MI, the findings and study types of these studies have been inconsistent. The goal of this meta-analysis was to critically assess the strength of the association between MI and periodontitis in case-control studies.

**Methods:** PubMed and the Cochrane Library were searched for eligible case-control studies reporting relevant parameters that compared periodontal status between MI and control subjects. The odds ratios (ORs) and 95% confidence intervals (CIs) from each study were pooled to estimate the strength of the association between MI and periodontitis. The mean differences and 95% CIs for periodontal-related parameters were calculated to determine their overall effects.

**Results:** Seventeen studies including a total of 3456 MI patients and 3875 non-MI control subjects were included. The pooled OR for the association between MI and periodontitis was 2.531 (95% CI: 1.927–3.324). The mean differences (95% CIs) for clinical attachment loss, probing depth, bleeding on probing, plaque index, and the number of missing teeth were 1.000 (0.726–1.247), 1.209 (0.538–1.880), 0.342 (0.129–0.555), 0.383 (0.205–0.560), and 4.122 (2.012–6.232), respectively.

**Conclusion:** With the current evidence, the results support the presence of a significant association between MI and periodontitis. Moreover, MI patients had worse periodontal and oral hygiene status and fewer teeth than did control subjects. More high-quality and well-designed studies focusing on the casual relationship between MI and periodontitis should be conducted in the future.

## Introduction

Myocardial infarction (MI), one type of cardiovascular disease (CVD), is caused by coronary artery occlusion resulting from atherosclerotic plaques. MI events can cause necrosis and apoptosis in a large number of myocardial cells and a severe reduction in the number of myocardial cells, which can directly lead to heart dysfunction and failure (He et al., 2016). Currently, CVD and subsequent myocardial infarctions remain leading causes of morbidity and mortality worldwide (Ferrero et al., 2014; GBD 2013 Mortality and Causes of Death Collaborators, 2015; Baron et al., 2016; Mozaffarian et al., 2016). The World Bank estimates that by 2030, 23 million Chinese patients will experience acute MI annually, which poses a serious challenge to the nation's healthcare system (Zhang et al., 2015; Tao et al., 2016). Therefore, it is of great importance to explore relevant risk factors and propose useful methods for the prevention and treatment of MI.

Periodontitis is a chronic inflammatory disease that damages the soft and hard tissue supporting the teeth, which results in loss of connective tissue attachment, erosion of the alveolar bone, and tooth loss (Lundmark et al., 2015). In the adult USA population, nearly half of individuals aged >30 years have some periodontitis, and almost 10% of the population has severe periodontitis (Eke et al., 2012, 2015, 2016a,b). There is growing evidence suggesting that periodontitis is associated with increased MI risk (Wożakowska-Kapłon et al., 2013; Kodovazenitis et al., 2014; Noguchi et al., 2015; Rydén et al., 2016). Periodontitis shares some common risk factors with MI, such as diabetes and smoking (Kjellström et al., 2016). In addition, growing literature indicates that local/systemic inflammation caused by periodontitis contributes to the risk of CVD, including MI events (De Nardin, 2001; Shrihari, 2012). An *in vivo* study showed that the periodontal pathogens could induce myocarditis and/or myocardial infarction in mice (Akamatsu et al., 2011).

Despite rapid growth in the number of studies on the possible etiological role of periodontal disease in the pathology of MI, this issue has not been resolved, and the findings of these studies have been inconsistent (Hujoel et al., 2000; Andriankaja et al., 2006; Dietrich et al., 2008; Sidhu, 2016). While the majority of publications have suggested an association between periodontitis and MI, several epidemiologic studies have found no such relationship (Hujoel et al., 2000; Howell et al., 2001). One systematic review (Sidhu, 2016) concluded that no relationship between these two conditions had been precisely replicated or verified, and therefore insufficient evidence was available to justify the hypothesis that periodontal interventions could prevent the onset or progression of MI events; the conclusion of that review was similar to that of the American Heart Association (AHA) (Lockhart et al., 2012). In addition, despite the existence of several systematic reviews and meta-analyses evaluating the association between periodontitis and CVD (Janket et al., 2003; Blaizot et al., 2009), specialized meta-analyses that quantitatively assess the presence and strength of the association between MI and periodontitis are still lacking.

Several different types of studies have been used to investigate the association between periodontitis and MI, including cross-sectional studies, case-control studies, and prospective and retrospective cohort studies. The pooling of data from studies using different designs has led to significant structural and methodological variation in published system reviews and meta-analysis and, therefore, increased heterogeneity (Kelly et al., 2013). To facilitate the determination of uniform statistical indicators and obtain more accurate and credible results, it is preferable to collect and merge data from the same type of research study. Therefore, the goal of this meta-analysis was to identify all related case-control studies investigating the association between periodontitis and MI and critically evaluate them to assess the strength of the association between MI and periodontitis. These results will provide clinicians and patients with better evidence-based evaluations and recommendations.

## Methods

### Focused topic

The main topics we focused on were as follows: 1. the presence and strength of the association between MI and periodontitis in case-control studies, and 2. whether the periodontal status of MI patients was worse than that of the control subjects.

### Literature-search strategy

We searched PubMed for related studies in August 2016, and the language of the search was restricted to English. Then, we searched the Cochrane Library without restrictions on eligible studies. The combination of the following key words was used: “periodontitis,” “periodontal disease,” and “myocardial infarction.” In addition, the reference lists provided in the related articles and reviews were also considered.

### Inclusion and exclusion criteria

In this meta-analysis, all available case-control studies with relevant parameters reported that compared periodontal status between MI and non-MI patients were included. Studies with data that could be extracted or calculated for pooling were also included. The exclusion criteria were (1) animal studies or *in vitro* studies; (2) reviews, letters, case reports or comments; (3) studies without data that could be extracted; and (4) studies without a case-control study design.

Briefly, according to the aforementioned inclusion criteria, the potential studies were selected as follows. First, after eliminating duplicate articles, irrelevant records were excluded by reading the titles and abstracts. Then, full-texts of the potential studies were scanned, and only the studies meeting the inclusion criteria were ultimately included in our meta-analysis. These results were independently assessed by two reviewers (SQ and ZB.), and any disagreement was resolved through discussion with a third reviewer (XJ).

### Data extraction and quality assessment

The following information was extracted from each included study: the study ID (first author and year of publication), county, type of dental surgery, characteristics of the subjects (including the number of patients in both groups and patient age and gender), value of odds ratios (ORs), adjusted or matched factors, periodontal parameters (i.e., clinical attachment loss (CAL), probing depth (PD), bleeding on probing (BOP), plaque index (PI), number of missing teeth), methods of dental examination, and brief conclusions. This process was independently performed by two reviewers (SQ and XJ).

The Newcastle-Ottawa Scale (NOS) was used to assess the quality of the included studies by two reviewers (HN and CC). In this assessment tool, the study selection, comparability, and exposure are used to appraise the methodological quality of the included studies, with a maximum of 9 points for each study. (GA Wells)^1^ NOS scores of 1–3, 4–6, and 7–9 indicated low, moderate, and high study quality, respectively.

### Statistical analysis

Statistical analyses were performed using the Comprehensive Meta-Analysis software package (Version 2.0; Biostat). OR values and their 95% confidence intervals (CIs) from each study were pooled to estimate the strength of the association between MI and periodontitis. Subgroup analysis were performed to determine whether particular characteristics of studies (quality, regions, method of periodontal assessment and number of adjustment for confounding factors) were associated with the value of the overall OR and 95% CI. Sensitivity analysis and cumulative analysis was performed to analysis the stability of the pooled results.

Because all the parameters used for evaluating periodontal status in this meta-analysis were continuous, mean differences, standard deviations and 95% CIs were calculated for individual trials and the overall effects.

Statistical heterogeneity between studies was tested using *I*^2^ statistics. A fixed effects model was used if the *I*^2^ <50%. *I*^2^ > 50% was considered to be substantial heterogeneity and the random effects was used.

## Results

### Summary of the included studies

**Figure 1:**
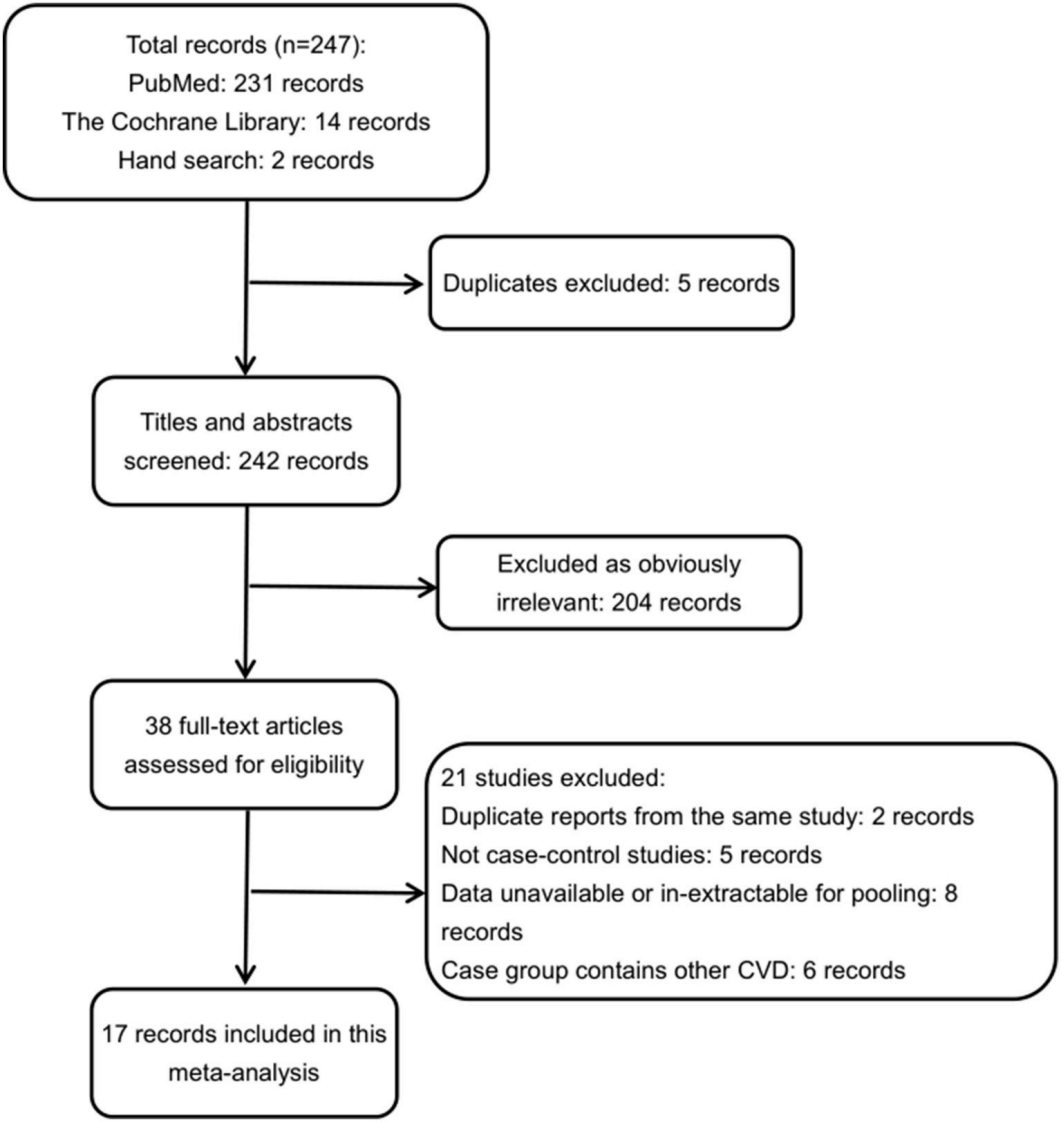
**Flow diagram for the selection of studies**. CVD, cardiovascular disease.

In total, 247 potentially relevant articles were identified through our search strategies. Of these studies, 209 were excluded according to the aforementioned inclusion criteria after a review of study titles and abstracts, including reviews, comments, and animal studies. After their exclusion, 38 articles were selected for further full-text review. Eventually, 17 studies (Emingil et al., 2000; Rutger Persson et al., 2003; Deliargyris et al., 2004; Renvert et al., 2004; Cueto et al., 2005; Andriankaja et al., 2006, 2007; Kaisare et al., 2007; Stein et al., 2009; Willershausen et al., 2009, 2014; Holmlund et al., 2011; Kodovazenitis et al., 2011, 2014; Khosravi Samani et al., 2013; Wożakowska-Kapłon et al., 2013; Rydén et al., 2016) were included in our meta-analysis. A flow diagram of search process and results is shown in Figure 1.

The publication dates of the 17 included case-control studies ranged from 2000 to 2016. Thirteen studies were conducted in European countries (Emingil et al., 2000; Rutger Persson et al., 2003; Deliargyris et al., 2004; Renvert et al., 2004; Cueto et al., 2005; Stein et al., 2009; Willershausen et al., 2009, 2014; Holmlund et al., 2011; Kodovazenitis et al., 2011, 2014; Wożakowska-Kapłon et al., 2013; Rydén et al., 2016), 2 studies were conducted in USA (Andriankaja et al., 2006, 2007) and 2 were conducted in Asian countries (Kaisare et al., 2007; Khosravi Samani et al., 2013). Within the included studies, a total of 3456 MI patients and 3875 non-MI control subjects had been evaluated. Moreover, there were 34 edentulous patients included in the MI groups of these studies and 7 edentulous patients included in the control groups of these studies; these patients were excluded from the all of the following comprehensive analysis. The characteristics of included studies and patients are shown in Table 1. The conclusions of the included studies were consistent: almost all of the studies provided evidence of an association between periodontitis and MI and worsened periodontal status in MI patients. The methods of periodontal evaluation and main conclusions of the included studies can be found in the Table S1.

**Table 1:** **Characteristics of included studies**.

**Study ID (First author, Year)**	**Country**	**Case (MI)**		**Control**		**OR value (95%CI)**	**Adjusted or matched factors**	**NOS score**
		**Number (male/female)**	**Age (mean ± SD)**	**Number (male/female)**	**Age (mean ± SD)**			
Rydén et al., 2016	Sweden	805(654/151)	62±8	805(654/151)	62±8	1.28 (1.03–1.60)	Age, gender, smoking, medical history, habits, diabetes, education, marital status	9
Kodovazenitis et al., 2014	Greece	204(156/48)	64.7±12.9	102(62/40)	64.2±10.1	2.45 (1.35–4.59)	Age, gender, hypertension, total cholesterol, smoking	6
Willershausen et al., 2014	Germany	248(201/47)	62.3±10.1	249(179/70)	63.5±10.5	1.146 (0.790–1.663)	Age, gender	6
Wożakowska-Kapłon et al., 2013	Poland	112(85/27)	53.4±6.5	67(43/24)	54.6±9.4	1.87 (1.41–2.48)	Age, gender, smoking	6
Khosravi Samani et al., 2013	Iran	60	54.97±9.68	63	55.89±11.9	8.79 (2.36–32.66)	—	4
Kodovazenitis et al., 2011	Greece	47(25/22)	69.6±11.9	40(20/20)	67.9±10.9	2.926 (1.071–7.998)	Age, gender	6
Holmlund et al., 2011	Sweden	100(79/21)	57.1±5.5	100(80/20)	57.9±5.2	4.61 (1.52–13.94)	Age, gender, education	6
Willershausen et al., 2009	Germany	125 (106/19)	61.8±10.4	125(97/32)	63.4±10.7	3.572 (1.881–6.785)	Gender, age, ethnicity, smoking	5
Stein et al., 2009	Germany	54(50/4)	50.8±6.3	50(47/3)	51.7±6.5	4.014 (1.738–9.272)	Age, gender	7
Andriankaja et al., 2007	USA	574(443/131)	54.96±8.95	887(385/502)	55.14±10.04	1.443 (1.252–1.663)	Age, BMI, physical activity, hypertension, cholesterol, diabetes, total pack-years of cigarette smoking	7
Kaisare et al., 2007	India	250(143/107)	55.5±9.8	250(144/106)	55.4±8.2	1.062	Age, gender, education, marital status, occupation	5
Andriankaja et al., 2006	USA	537(414/123)	54.6±8.5	800(351/449)	55.0±0.0	2.08 (1.51–2.87)	Age, gender, hypertension, cholesterol, diabetes, total pack-years of cigarette smoking	7
Cueto et al., 2005	Spain	72(50/22)	62.5±9.9	77(39/38)	58.5±10.2	3.31 (1.42–7.71)	Age, gender, diabetes, hypertension, smoking, cholesterol, exercise	6
Renvert et al., 2004	Sweden	88	62.7±9.1	80	62.7±9.1	7.67 (1.13–51.92)	Age, gender, social group	6
Deliargyris et al., 2004	Greece	40(29/11)	60±11	40(31/9)	64±3	4.26 (1.53–11.89)	Age, gender, race	7
Rutger Persson et al., 2003	Sweden	80	63.4±8.9	80	61.9±9.1	14.1 (5.8–34.4)	Age, gender	8
Emingil et al., 2000	Turkey	60(50/10)	53.81±9.50	60(42/18)	58.53±11.69	—	—	6

*MI, myocardial infarction; OR, odds ratio; CI, confidence interval; SD, standard deviation; NOS, Newcastle-Ottawa Scale; BMI, Body Mass Index*.

The results of the methodological quality assessment of the 17 included studies performed using the NOS are also shown in Table 1. Six studies (Rutger Persson et al., 2003; Deliargyris et al., 2004; Andriankaja et al., 2006, 2007; Stein et al., 2009; Rydén et al., 2016) scored more than 7 points and were considered to be of high quality. Eleven studies (Emingil et al., 2000; Renvert et al., 2004; Cueto et al., 2005; Kaisare et al., 2007; Willershausen et al., 2009, 2014; Holmlund et al., 2011; Kodovazenitis et al., 2011, 2014; Khosravi Samani et al., 2013; Wożakowska-Kapłon et al., 2013) scored less than 6 points but more than 4 points, and these studies were considered to be of moderate quality. The mean score (6.29) was higher than 6, suggesting that the included studies had acceptable quality.

### Meta-analysis

#### Overall OR

**Figure 2:**
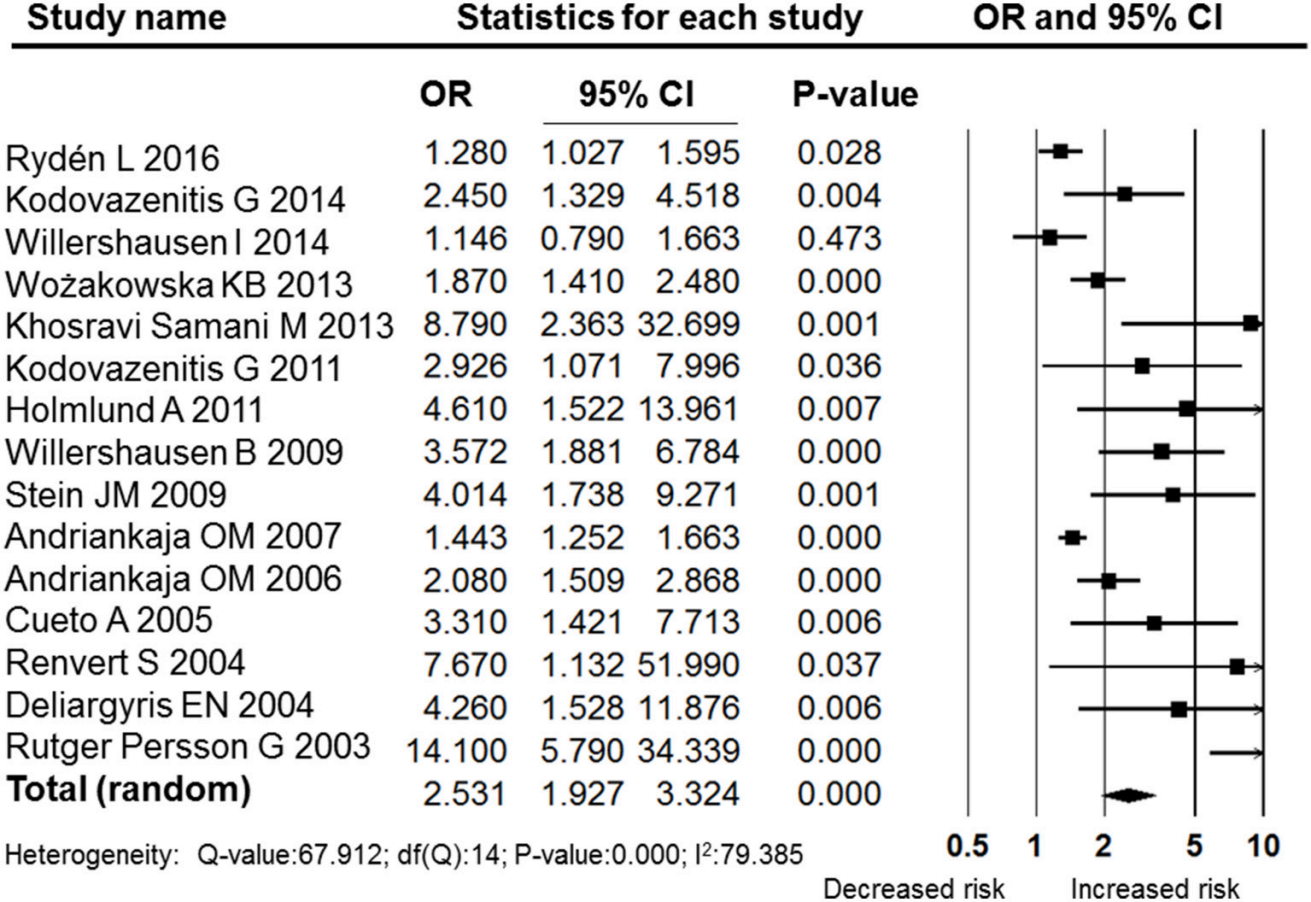
**Forest plot of the overall analysis of the association between MI and periodontitis**. OR, odds ratio; CI, confidence interval.

A meta-analysis of the ORs assessing the odds of periodontitis between case (MI) and control (non-MI) patients was performed using data from the 15 included studies that reported or had data available to calculate the OR and 95% CI. The pooled OR for the case versus control subjects was 2.531 (95% CI: 1.927–3.324, Figure 2), and this difference was statistically significant (*P* = 0.000, Figure 2). This result showed that MI patients had a higher odds of periodontitis than did the control subjects. However, the heterogeneity between the included studies was high (*I*^2^: 79.385); hence, a random effects model was selected.

**Figure 3:**
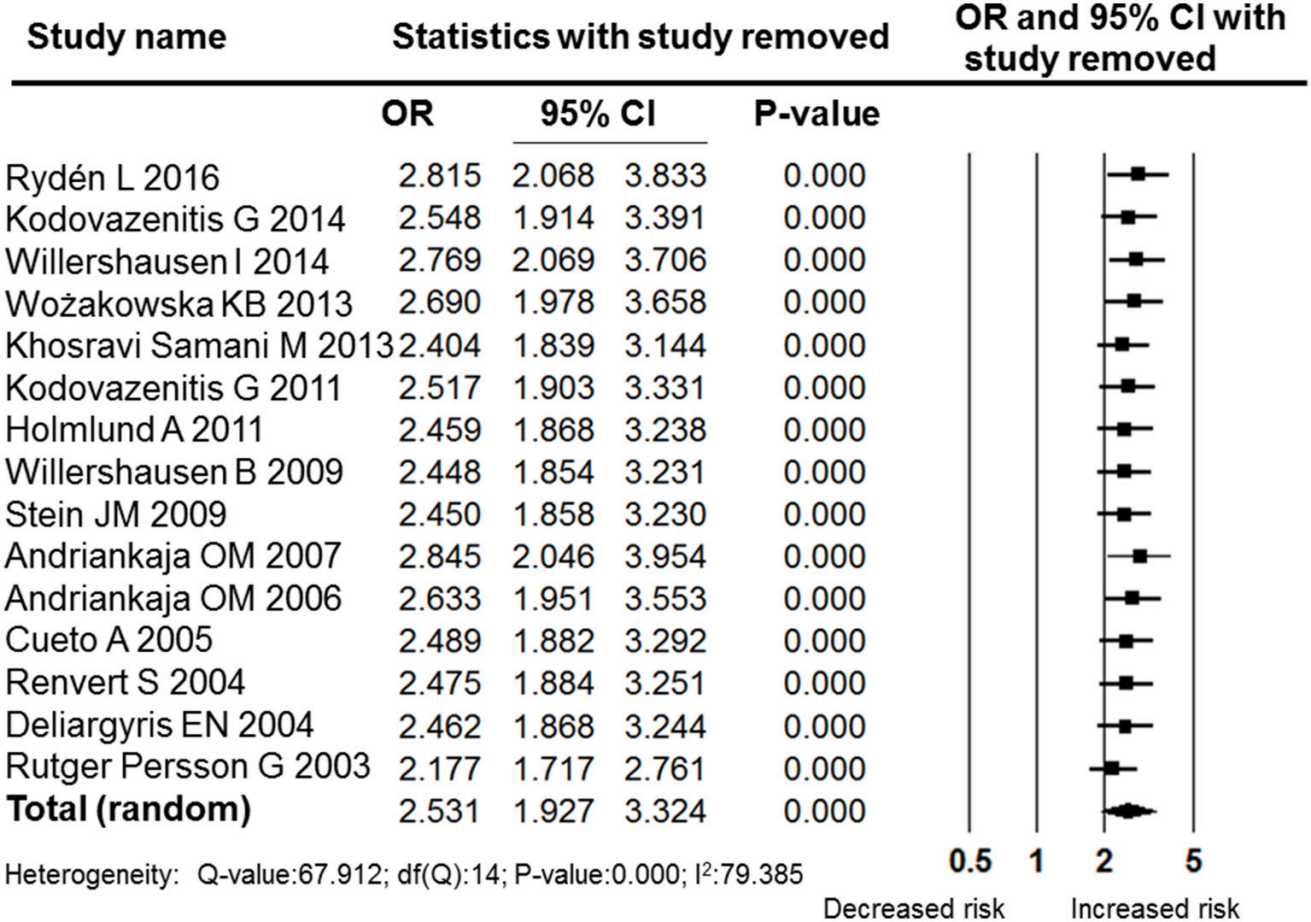
**Forest plot of the sensitivity analysis performed by sequential removing single studies**. OR, odds ratio; CI, confidence interval.

**Figure 4:**
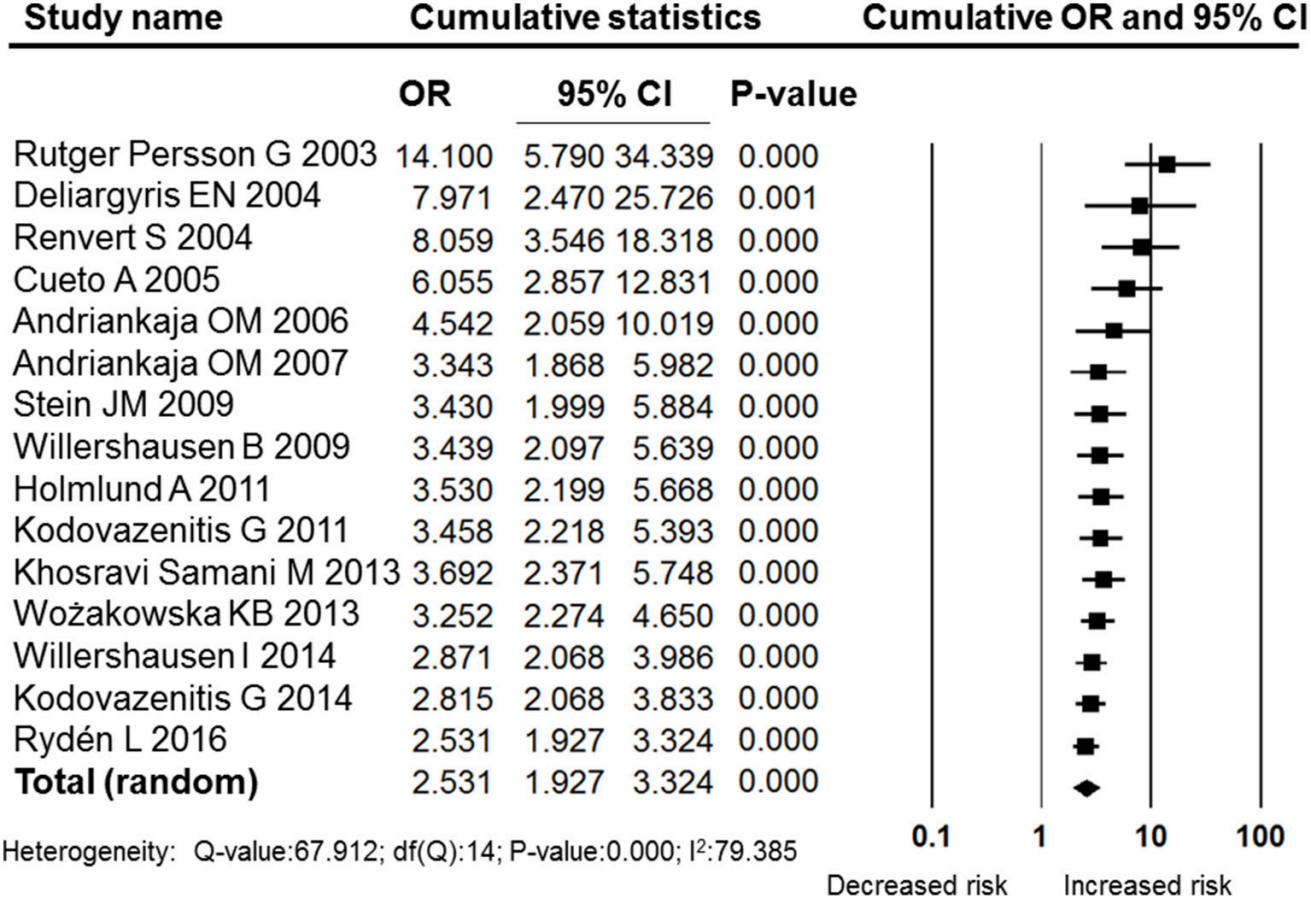
**Forest plot of the cumulative meta-analysis by publication year**. OR, odds ratio, CI, confidence interval.

Despite the high level heterogeneity, the sensitivity analysis performed by sequentially removing individual studies did not change the results (Figure 3). Furthermore, a cumulative analysis performed by the order of publication date revealed stable and statistically significant results (Figure 4) that indicated a significant association between MI and periodontitis. Subgroup analysis according to quality, region, method of periodontal assessment and number of confounding factors adjusted for in the model all revealed significant associations between MI and periodontitis (Table 2). Moreover, analysis in each subgroup also revealed high heterogeneity; therefore, subgroup analyses were also performed using random effects models.

**Table 2:** **Statistics for subgroup analyses**.

**Subgroup**	**No. of studies**	**Statistics for each subgroup**		
		**OR (95%CI)**	***P*-value**	***I*^2^-value**
All studies	15	2.531(1.927–3.324)	0.000	79.385
**QUALITY**				
High (7–9)	6	2.474 (1.634–3.748)	0.000	87.318
Moderate (4–6)	9	2.655 (1.800–3.916)	0.000	65.589
**REGIONS**				
European countries	12	2.844 (1.954–4.139)	0.000	79.974
USA	2	1.682 (1.181–2.396)	0.000	76.001
Asian countries	1	8.790 (2.363–32.699)	0.001	0.000
**ASSESSMENT OF PERIODONTAL DISEASE**				
With radiological examination	7	2.740 (1.631–4.605)	0.000	86.502
Without radiological examination	8	2.520 (1.776–3.576)	0.000	70.161
**NUMBER OF CONFOUNDING FACTORS**				
≥5	5	1.698 (1.329–2.170)	0.000	66.903
<5	10	3.657 (2.219–6.027)	0.000	79.306

*OR, odds ratio; CI, confidence interval*.

#### CAL

**Figure 5:**
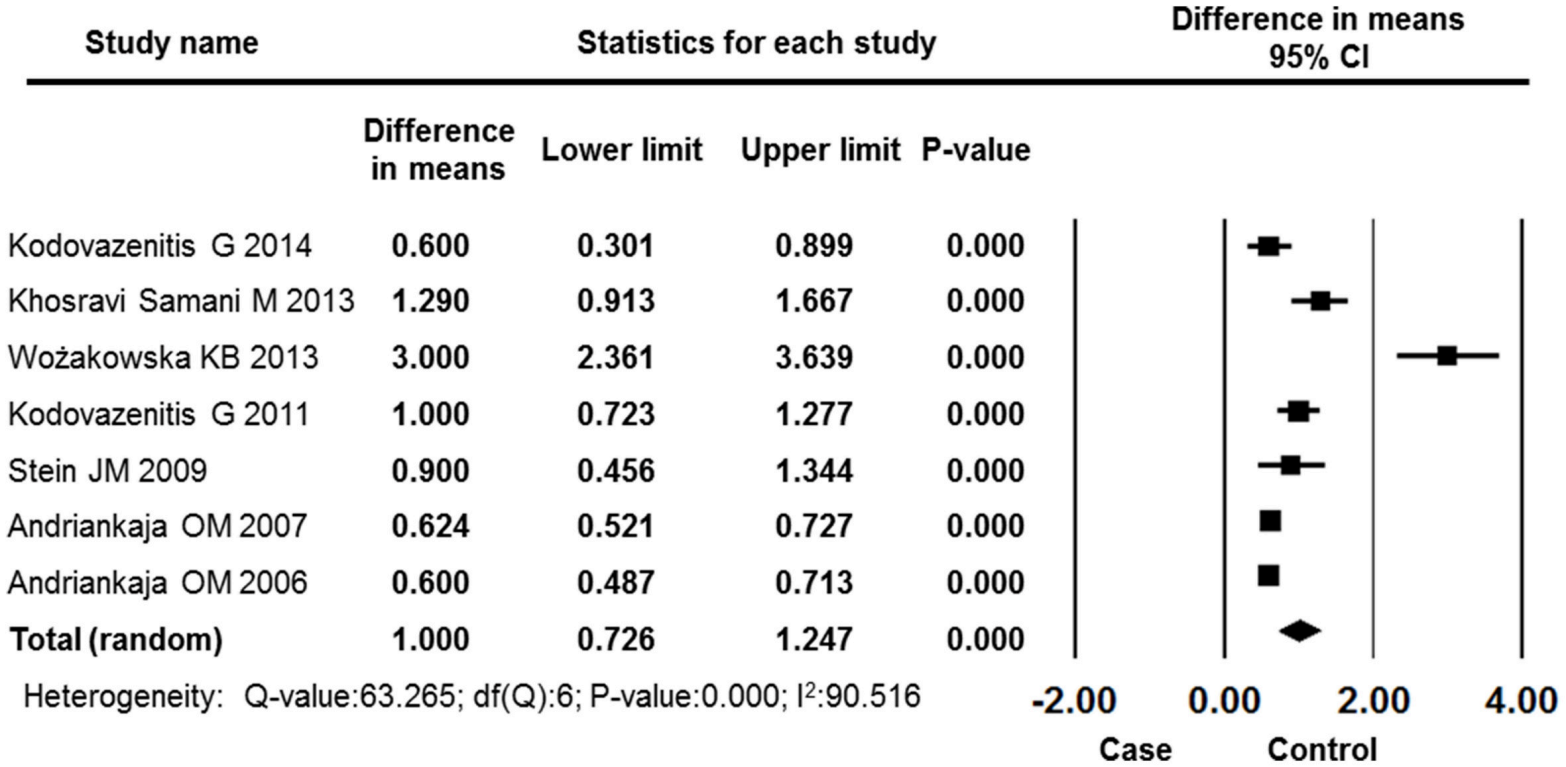
**Forest plot of the mean difference in CAL between the case and control groups**. CI, confidence interval.

Seven studies reported CAL results for both groups. The overall effects showed patients in the MI group suffered more clinical attachment loss than did control group patients, and this difference was statistically significant (difference in means: 1.000 mm, 95% CI: 0.726–1.247, *P* = 0.000, random effects model, Figure 5).

#### PD

**Figure 6:**
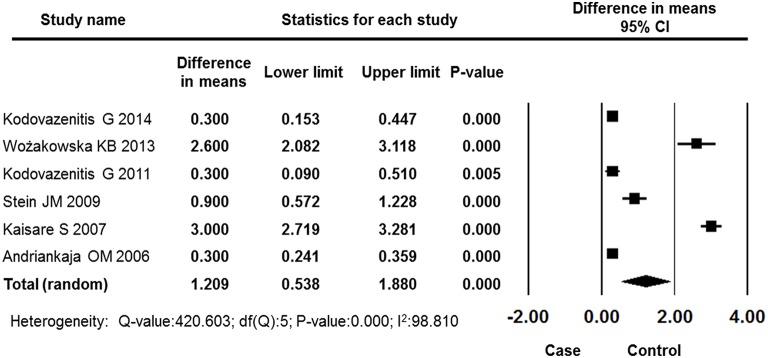
**Forest plot of the mean difference in PD between the case and control groups**. CI, confidence interval.

Six studies reported PD data for the MI and control groups. The results of meta-analysis revealed that PD was deeper in the MI group than in the control group, and the difference in means was 1.209 mm (95% CI: 0.538–1.880, *P* = 0.000, random effect model, Figure 6).

#### BOP

**Figure 7:**
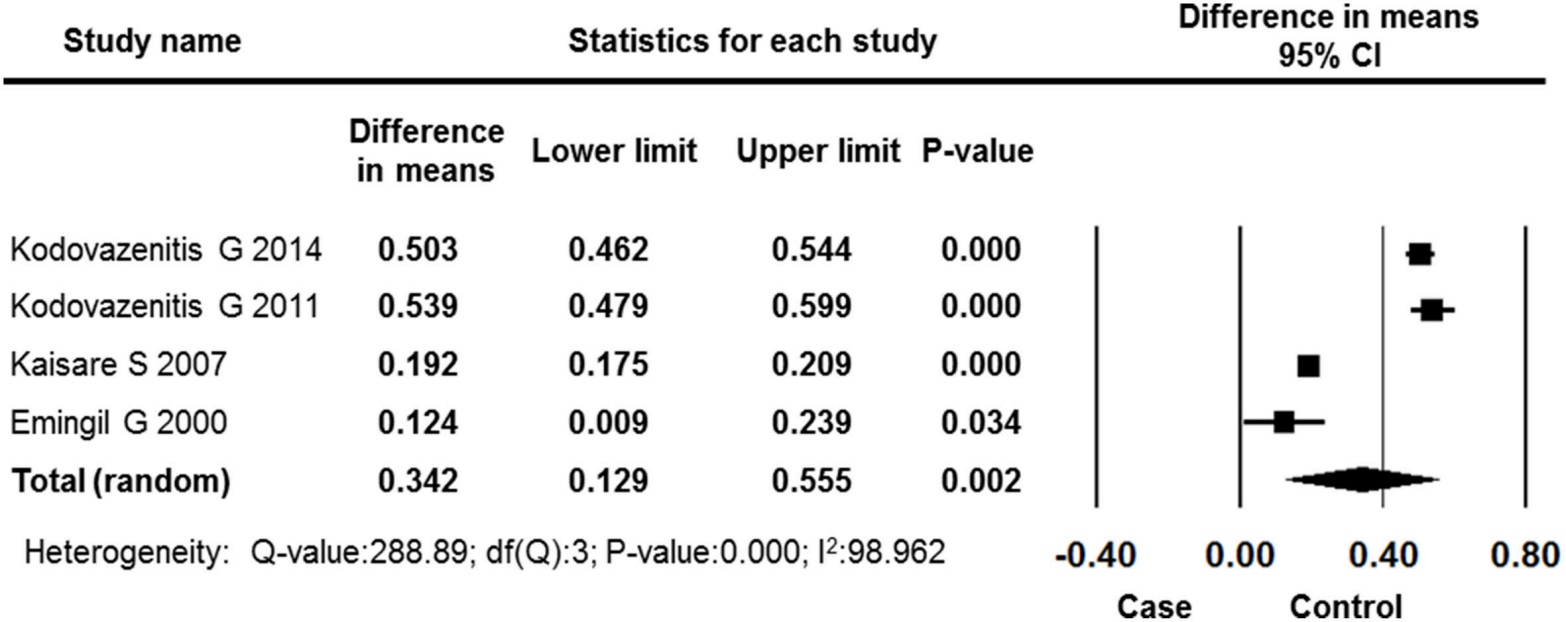
**Forest plot of the mean difference in BOP between the case and control groups**. CI, confidence interval.

Four studies reported BOP data for the MI and control groups. The overall effects showed a significantly higher level of BOP among MI patients compared with control subjects (difference in means: 0.342, 95% CI: 0.129–0.555, *P* = 0.002, random effects model, Figure 7).

#### PI

**Figure 8:**
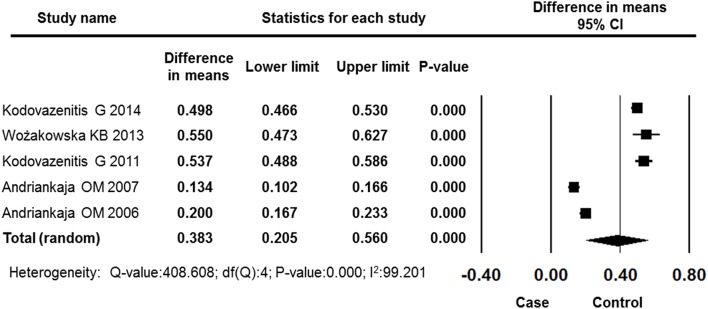
**Forest plot of the mean difference in PI between the case and control groups**. CI, confidence interval.

Five studies reported PI data for the MI and control groups. The overall effects showed a significantly higher level of plaque among MI patients compared with control subjects (difference in means: 0.383, 95% CI: 0.205–0.560, *P* = 0.000, random effects model, Figure 8).

#### Missing teeth

**Figure 9:**
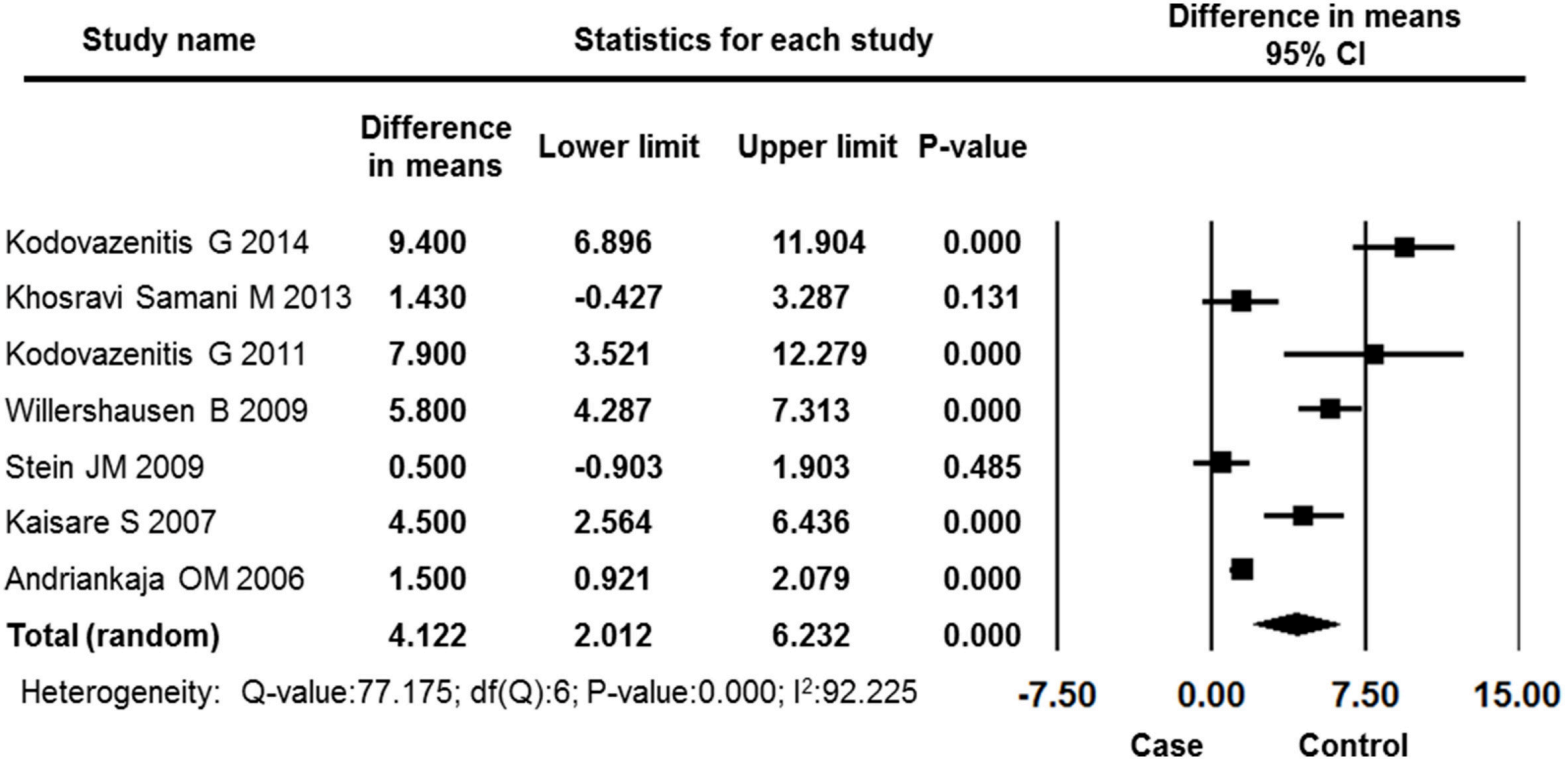
**Forest plot of the mean difference in missing teeth between the case and control groups**. CI, confidence interval.

Seven studies counted the number of missing teeth in the MI and control groups. The results of the meta-analysis showed that MI patients lost more teeth compared with control group patients, and this difference was statistically significant (difference in means: 4.122, 95% CI: 2.012–6.232, *P* = 0.000, random effects model, Figure 9).

## Discussion

At present, the majority of studies focusing on the association between periodontitis and MI have been observational studies. Despite the fact that is has been suggested that randomized control trials (RCT) provide a higher-quality of evidence, this type of study cannot be used to answer all clinical questions for ethical, methodological, or logistic reasons (Guyatt et al., 2008; Blaizot et al., 2009). In this meta-analysis, to reduce structural and methodological variation and obtain more accurate results, the included studies were confined to those using a case-control methodology. Analysis of data from the 17 case-control studies included in our meta-analysis revealed that compared with the control subjects, MI patients had higher odds of periodontitis and more serious periodontitis, indicating there was a significant association between MI and periodontitis. Moreover, the MI patients suffered from worse periodontal status and more tooth loss.

The pooled OR was 2.531 (95% CI: 1.927–3.324), indicating that periodontitis was significantly associated with MI. Our results were similar to the findings of another meta-analysis that focused on the association between periodontal diseases and cardiovascular events; in that study, the pooled OR calculated using data from the 22 case-control and cross-sectional studies were 2.35 (Blaizot et al., 2009). Both their and our meta-analysis showed high statistical heterogeneity among included studies. In this research, the *I*^2^-value was 79.385, and a random effects model was adopted. However, the sensitivity analysis performed by sequentially removing individual studies did not change the results (Figure 3). Furthermore, the cumulative analysis performed by the order of publication date revealed stable and statistically significant results (Figure 4). In the subgroup analyses, the included studies were grouped according to quality, region, method of periodontal assessment, and number of confounding factors adjusted for in the models. Those results of all the subgroup analyses revealed significant associations between MI and periodontitis (Table 2). Moreover, analysis performed within each subgroup also revealed high levels of heterogeneity.

Based on the current literature, the observed heterogeneity may have resulted from the following aspects. First, different diagnostic criteria and methods of periodontitis assessment were used. However, some researchers found that the association between periodontitis and MI was consistent regardless of the different measurements and case definitions of periodontitis used (Andriankaja et al., 2006; Kodovazenitis et al., 2014). It is undeniable that different criteria and methods will lead to variation in the outcome measures. Second, different types of control subjects were included. Among the included studies, healthy subjects (Kodovazenitis et al., 2014; Willershausen et al., 2014), trauma patients (Cueto et al., 2005), or the CVD patients without MI (Kaisare et al., 2007; Wożakowska-Kapłon et al., 2013) were recruited as control subjects. Third, incomplete and differential adjustment for confounding factors may have played a role. Some factors can affect both MI and periodontitis independently, such as tobacco, diabetes, and age (Guiglia et al., 2008; Blaizot et al., 2009; Kodovazenitis et al., 2014). The included studies matched or adjusted for different types and numbers of the confounding factors. In this meta-analysis, the number of the confounding factors considered in the included studies ranged from 0 to 8. Forth, myocardial infarction is a medical emergency, and only the patients who survived an MI event (excluding those patients died of MI) had been included in these studies. These factors are not all of the reasons that heterogeneity was observed; however, they may have played a critical role in contributing to the high level heterogeneity identified among these observational studies.

Through comprehensive analysis of the periodontal parameters, the overall effects showed that periodontal status and oral hygiene were worse in MI patients compared to control subjects. The periodontal measurements CAL and PD have been well established as reflections of periodontal disease in both clinical research and clinical practice (Machtei et al., 1993; Andriankaja et al., 2006; Kodovazenitis et al., 2014). In addition, these two factors reflect both past periodontitis and recent inflammation in patients. This meta-analysis found that MI patients had more attachment loss and deeper periodontal pockets. The differences in the means values of CAL and PD between MI and control subjects were 1.000 and 1.209 mm, respectively. Moreover, in the MI patients, a significantly higher level of plaque and BOP were identified compared with control subjects, indicating poor oral hygiene in MI patients. However, the reason that MI patients had a higher level of BOP and dental plaque might also be that they had longer stays in the intensive care unit and hospital during which they may not or could not have maintained their daily oral hygiene (Rutger Persson et al., 2003; Renvert et al., 2004). Furthermore, the overall effect revealed that the MI patients had lost more than 4 teeth (difference in means: 4.122, 95% CI: 2.012–6.232, *P* = 0.000) more than the control subjects. While tooth loss can be caused by many reasons, the greater extent of tooth loss in this population somewhat reflects a previous accumulation of oral inflammation, (Liljestrand et al., 2015) and many studies have determined there to be an association between MI status and the number of missing teeth (Andriankaja et al., 2006; Stein et al., 2009; Kodovazenitis et al., 2011, 2014).

This meta-analysis showed that there was a significant association between MI and periodontitis. While there is still a lack of relevant evidence for the causal relationship between MI and periodontitis (Blaizot et al., 2009; Stewart and West, 2016), it is undeniable that this associations is of great importance because of the potential effect that preventing or treating periodontal disease could have on reducing the risk of MI. In the long-term, the achievement of a sustained improvement in periodontal health and reduction in the risk of MI or relevant complications, rather than a single treatment, have been recommended (Stewart and West, 2016). However, the AHA has noted that although periodontal interventions result in a reduction in systemic inflammation and endothelial dysfunction in short-term studies, there is no evidence that they prevent atherosclerotic vascular disease or modify its outcomes (Lockhart et al., 2012). In addition, some researchers have concluded that insufficient evidence is available to justify the hypothesis that periodontal interventions can prevent the onset or progression of acute myocardial events (Li et al., 2014; Henschel and Keenan, 2015; Sidhu, 2016). Therefore, more clinical research to evaluate the effectiveness of interventions for periodontitis on the reduction of MI events is needed in future.

To our knowledge, this is the first meta-analysis to specifically estimate the association between MI and periodontitis using quantitative analysis. We included only case-control studies to reduce structural and methodological variation. We not only assessed the association between MI and periodontitis by pooling the OR values reported by each study but also examined relevant periodontal parameters to evaluate periodontal and oral hygiene status. Moreover, sensitivity and cumulative analyses were performed to determine the stability of the pooled results. However, our meta-analysis has three limitations. First, heterogeneity among the included studies was high, both in the overall effect analysis and the subgroup analyses. Second, the search strategy was confined to English-language studies, which might have introduced a selection bias into this meta-analysis. Third, due to the limited number of studies analyzing each of the periodontal parameters, sensitivity, cumulative and subgroup analyses were not performed.

In summary and in consideration of the strengths and limitations of this study, the pooled data evaluated in this meta-analysis support the conclusion that MI patients had higher odds of periodontitis and more serious periodontitis than did controls, and there was a significant association between MI and periodontitis. At the same time, MI patients had worse periodontal and oral hygiene status and fewer teeth than did control subjects. More importantly, more high-quality and well-designed studies focused on the casual relationship between MI and periodontitis should be conducted. In addition, studies focused on the effect of interventions for periodontitis on the reduction of MI events are also needed in the future.

## Author contributions

The sections on literature research, study selection and data extraction were completed by QS and BZ; the section on risk of bias evaluation and data analysis were completed by NH and CC; QS drafted the manuscript and BZ, JX helped to revise the manuscript. HL and JX are the corresponding authors, and they undertook the work of designing this meta-analysis, coordinating and helping to draft the manuscript. All authors read and approved the final manuscript.

## Funding

This study was supported in part by grants from the National High Technology Research and Development Program (“863” Program) of China (2015AA033502) and the National Natural Science Foundation of China (NO. 81271180, 81500861).

### Conflict of interest statement

The authors declare that the research was conducted in the absence of any commercial or financial relationships that could be construed as a potential conflict of interest. The reviewer PP and handling Editor declared their shared affiliation, and the handling Editor states that the process nevertheless met the standards of a fair and objective review.

## Footnotes

^1^Ga Wells, B. S., O'connell, D., Peterson, J., Welch, V., Losos, M., and Tugwell, P. *The Newcastle-Ottawa Scale (NOS) for Assessing the Quality of Non-randomized Studies in Meta-Analyses* [Online]. Available online at: http://www.ohri.ca/programs/clinical_epidemiology/oxford.asp [Accessed].

## References

[B2] Akamatsu Y. Yamamoto T. Yamamoto K. Oseko F. Kanamura N. Imanishi J.. (2011). Porphyromonas gingivalis induces myocarditis and/or myocardial infarction in mice and IL-17A is involved in pathogenesis of these diseases. Arch. Oral. Biol. 56, 1290–1298. 10.1016/j.archoralbio.2011.05.012 21683342

[B3] Andriankaja O. M. Genco R. J. Dorn J. Dmochowski J. Hovey K. Falkner K. L.. (2006). The use of different measurements and definitions of periodontal disease in the study of the association between periodontal disease and risk of myocardial infarction. J. Periodontol. 77, 1067–1073. 10.1902/jop.2006.050276 16734583

[B4] Andriankaja O. M. Genco R. J. Dorn J. Dmochowski J. Hovey K. Falkner K. L.. (2007). Periodontal disease and risk of myocardial infarction: the role of gender and smoking. Eur. J. Epidemiol. 22, 699–705. 10.1007/s10654-007-9166-6 17828467

[B5] Baron T. Hambraeus K. Sundström J. Erlinge D. Jernberg T. Lindahl B. (2016). Impact on long-term mortality of presence of obstructive coronary artery disease and classification of myocardial infarction. Am. J. Med. 129, 398–406. 10.1016/j.amjmed.2015.11.035 26763754

[B6] Blaizot A. Vergnes J. N. Nuwwareh S. Amar J. Sixou M. (2009). Periodontal diseases and cardiovascular events: meta-analysis of observational studies. Int. Dent. J. 59, 197–209. 10.1922/IDJ_2114Sixou13 19774803

[B7] Cueto A. Mesa F. Bravo M. Ocaña-Riola R. (2005). Periodontitis as risk factor for acute myocardial infarction. A case control study of Spanish adults. J. Periodontal. Res. 40, 36–42. 10.1111/j.1600-0765.2004.00766.x 15613077

[B8] Deliargyris E. N. Madianos P. N. Kadoma W. Marron I. Smith S. C. Jr. Beck J. D.. (2004). Periodontal disease in patients with acute myocardial infarction: prevalence and contribution to elevated C-reactive protein levels. Am. Heart J. 147, 1005–1009. 10.1016/j.ahj.2003.12.022 15199348

[B9] De Nardin E. (2001). The role of inflammatory and immunological mediators in periodontitis and cardiovascular disease. Ann. Periodontol. 6, 30–40. 10.1902/annals.2001.6.1.30 11887469

[B10] Dietrich T. Jimenez M. Krall Kaye E. A. Vokonas P. S. Garcia R. I. (2008). Age-dependent associations between chronic periodontitis/edentulism and risk of coronary heart disease. Circulation 117, 1668–1674. 10.1161/CIRCULATIONAHA.107.711507 18362228 PMC2582144

[B11] Eke P. I. Dye B. A. Wei L. Slade G. D. Thornton-Evans G. O. Borgnakke W. S.. (2015). Update on prevalence of periodontitis in adults in the United States: NHANES 2009 to 2012. J. Periodontol. 86, 611–622. 10.1902/jop.2015.140520 25688694 PMC4460825

[B12] Eke P. I. Dye B. A. Wei L. Thornton-Evans G. O. Genco R. J. (2012). Prevalence of periodontitis in adults in the United States: 2009 and 2010. J. Dent. Res. 91, 914–920. 10.1177/0022034512457373 22935673

[B13] Eke P. I. Wei L. Borgnakke W. S. Thornton-Evans G. Zhang X. Lu H.. (2016a). Periodontitis prevalence in adults >/ = 65 years of age, in the USA. Periodontology 2000 72, 76–95. 10.1111/prd.12145 27501492 PMC8223257

[B14] Eke P. I. Zhang X. Lu H. Wei L. Thornton-Evans G. Greenlund K. J.. (2016b). Predicting periodontitis at state and local levels in the United States. J. Dent. Res. 95, 515–522. 10.1177/0022034516629112 26848071 PMC6092742

[B15] Emingil G. Buduneli E. Aliyev A. Akilli A. Atilla G. (2000). Association between periodontal disease and acute myocardial infarction. J. Periodontol. 71, 1882–1886. 10.1902/jop.2000.71.12.1882 11156045

[B16] Ferrero J. M. Trenor B. Romero L. (2014). Multiscale computational analysis of the bioelectric consequences of myocardial ischaemia and infarction. Europace 16, 405–415. 10.1093/europace/eut405 24569895

[B1] GBD 2013 Mortality Causes of Death Collaborators (2015). Global, regional, and national age-sex specific all-cause and cause-specific mortality for 240 causes of death, 1990-2013: a systematic analysis for the Global Burden of Disease Study 2013. Lancet 385, 117–171. 10.1016/S0140-6736(14)61682-2 25530442 PMC4340604

[B17] Guiglia R. Lo Russo L. Coccia E. Di Liberto C. D'angelo M. Indovina G.. (2008). The association between periodontal diseases and cardiovascular diseases: a narrative review. Panminerva Med. 50, 327–337. 19078873

[B18] Guyatt G. H. Oxman A. D. Kunz R. Vist G. E. Falck-Ytter Y. Schunemann H. J. (2008). What is “quality of evidence” and why is it important to clinicians? BMJ 336, 995–998. 10.1136/bmj.39490.551019.BE 18456631 PMC2364804

[B19] He X.-M. Chen L. Luo J.-B. Feng X.-X. Zhang Y.-B. Chen Q.-J.. (2016). Effects of rhBNP after PCI on non-invasive hemodynamic in acute myocardial infarction patients with left heart failure. Asian Pac. J. Trop. Med. 9, 791–795. 10.1016/j.apjtm.2016.06.006 27569890

[B20] Henschel M. Keenan A. V. (2015). Insufficient evidence of effect of periodontal treatment on prevention or management of cardiovascular disease. Evid. Based Dent. 16, 17–18. 10.1038/sj.ebd.6401079 25909935

[B21] Holmlund A. Hedin M. Pussinen P. J. Lerner U. H. Lind L. (2011). Porphyromonas gingivalis (Pg) a possible link between impaired oral health and acute myocardial infarction. Int. J. Cardiol. 148, 148–153. 10.1016/j.ijcard.2009.10.034 19913930

[B22] Howell T. H. Ridker P. M. Ajani U. A. Hennekens C. H. Christen W. G. (2001). Periodontal disease and risk of subsequent cardiovascular disease in U.S. male physicians. J. Am. Coll. Cardiol. 37, 445–450. 10.1016/S0735-1097(00)01130-X 11216961

[B23] Hujoel P. P. Drangsholt M. Spiekerman C. Derouen T. A. (2000). Periodontal disease and coronary heart disease risk. JAMA 284, 1406–1410. 10.1001/jama.284.11.1406 10989403

[B24] Janket S. J. Baird A. E. Chuang S. K. Jones J. A. (2003). Meta-analysis of periodontal disease and risk of coronary heart disease and stroke. Oral Surg. Oral Med. Oral Pathol. Oral Radiol. Endod. 95, 559–569. 10.1067/moe.2003.107 12738947

[B25] Kaisare S. Rao J. Dubashi N. (2007). Periodontal disease as a risk factor for acute myocardial infarction. A case-control study in Goans highlighting a review of the literature. Br. Dent. J. 203, E5. discussion: 144–145. 10.1038/bdj.2007.690 17694042

[B26] Kelly J. T. Avila-Ortiz G. Allareddy V. Johnson G. K. Elangovan S. (2013). The association between periodontitis and coronary heart disease: a quality assessment of systematic reviews. J. Am. Dent. Assoc. 144, 371–379. 10.14219/jada.archive.2013.0130 23543691

[B27] Khosravi Samani M. Jalali F. Seyyed Ahadi S. M. Hoseini S. R. Dabbagh Sattari F. (2013). The relationship between acute myocardial infarction and periodontitis. Caspian J. Intern. Med. 4, 667–671. 24009957 PMC3755827

[B28] Kjellström B. Rydén L. Klinge B. Norhammar A. (2016). Periodontal disease - important to consider in cardiovascular disease prevention. Expert Rev. Cardiovasc. Ther. 14, 987–989. 10.1080/14779072.2016.1202112 27310921

[B29] Kodovazenitis G. Pitsavos C. Papadimitriou L. Deliargyris E. N. Vrotsos I. Stefanadis C.. (2011). Periodontal disease is associated with higher levels of C-reactive protein in non-diabetic, non-smoking acute myocardial infarction patients. J. Dent. 39, 849–854. 10.1016/j.jdent.2011.09.005 21946158

[B30] Kodovazenitis G. Pitsavos C. Papadimitriou L. Vrotsos I. A. Stefanadis C. Madianos P. N. (2014). Association between periodontitis and acute myocardial infarction: a case-control study of a nondiabetic population. J. Periodont. Res. 49, 246–252. 10.1111/jre.12101 23713486

[B31] Li C. Lv Z. Shi Z. Zhu Y. Wu Y. Li L.. (2014). Periodontal therapy for the management of cardiovascular disease in patients with chronic periodontitis. Cochrane Database Syst. Rev. Cd009197. 10.1002/14651858.CD009197.pub2 25123257

[B32] Liljestrand J. M. Havulinna A. S. Paju S. Männistö S. Salomaa V. Pussinen P. J. (2015). Missing teeth predict incident cardiovascular events, diabetes, and death. J. Dent. Res. 94, 1055–1062. 10.1177/0022034515586352 25991651

[B33] Lockhart P. B. Bolger A. F. Papapanou P. N. Osinbowale O. Trevisan M. Levison M. E.. (2012). Periodontal disease and atherosclerotic vascular disease: does the evidence support an independent association?: a scientific statement from the American Heart Association. Circulation 125, 2520–2544. 10.1161/CIR.0b013e31825719f3 22514251

[B34] Lundmark A. Davanian H. Båge T. Johannsen G. Koro C. Lundeberg J.. (2015). Transcriptome analysis reveals mucin 4 to be highly associated with periodontitis and identifies pleckstrin as a link to systemic diseases. Sci. Rep. 5:18475. 10.1038/srep18475 26686060 PMC4685297

[B35] Machtei E. E. Norderyd J. Koch G. Dunford R. Grossi S. Genco R. J. (1993). The rate of periodontal attachment loss in subjects with established periodontitis. J. Periodontol. 64, 713–718. 10.1902/jop.1993.64.8.713 8410609

[B36] Mozaffarian D. Benjamin E. J. Go A. S. Arnett D. K. Blaha M. J. Cushman M.. (2016). Heart disease and stroke statistics-2016 update: a report from the American Heart Association. Circulation 133, e38–360. 10.1161/CIR.0000000000000366 26673558

[B37] Noguchi S. Toyokawa S. Miyoshi Y. Suyama Y. Inoue K. Kobayashi Y. (2015). Five-year follow-up study of the association between periodontal disease and myocardial infarction among Japanese male workers: MY Health Up Study. J. Public Health (Oxf). 37, 605–611. 10.1093/pubmed/fdu076 25293424

[B38] Renvert S. Ohlsson O. Persson S. Lang N. P. Persson G. R. (2004). Analysis of periodontal risk profiles in adults with or without a history of myocardial infarction. J. Clin. Periodontol. 31, 19–24. 10.1111/j.0303-6979.2004.00431.x 15058370

[B39] Rutger Persson G. Ohlsson O. Pettersson T. Renvert S. (2003). Chronic periodontitis, a significant relationship with acute myocardial infarction. Eur. Heart J. 24, 2108–2115. 10.1016/j.ehj.2003.10.007 14643271

[B40] Rydén L. Buhlin K. Ekstrand E. De Faire U. Gustafsson A. Holmer J.. (2016). Periodontitis increases the risk of a first myocardial infarction: a report from the PAROKRANK study. Circulation 133, 576–583. 10.1161/CIRCULATIONAHA.115.020324 26762521

[B41] Shrihari T. G. (2012). Potential correlation between periodontitis and coronary heart disease–an overview. Gen. Dent. 60, 20–24. 22313976

[B42] Sidhu R. K. (2016). Association between acute myocardial infarction and periodontitis: a review of the literature. J. Int. Acad. Periodontol. 18, 23–33. 26764968

[B43] Stein J. M. Kuch B. Conrads G. Fickl S. Chrobot J. Schulz S.. (2009). Clinical periodontal and microbiologic parameters in patients with acute myocardial infarction. J. Periodontol. 80, 1581–1589. 10.1902/jop.2009.090170 19792846

[B44] Stewart R. West M. (2016). Increasing evidence for an association between periodontitis and cardiovascular disease. Circulation 133, 549–551. 10.1161/circulationaha.115.020869 26762522

[B45] Tao J. Wang Y. T. Abudoukelimu M. Yang Y. N. Li X. M. Xie X.. (2016). Association of genetic variations in the Wnt signaling pathway genes with myocardial infarction susceptibility in Chinese Han population. Oncotarget 7, 52740–52750. 10.18632/oncotarget.10401 27391264 PMC5288145

[B46] Willershausen B. Kasaj A. Willershausen I. Zahorka D. Briseno B. Blettner M.. (2009). Association between chronic dental infection and acute myocardial infarction. J. Endod. 35, 626–630. 10.1016/j.joen.2009.01.012 19410072

[B47] Willershausen I. Weyer V. Peter M. Weichert C. Kasaj A. Munzel T.. (2014). Association between chronic periodontal and apical inflammation and acute myocardial infarction. Odontology 102, 297–302. 10.1007/s10266-013-0112-7 23604464

[B48] Wożakowska-Kapłon B. Wlosowicz M. Gorczyca-Michta I. Górska R. (2013). Oral health status and the occurrence and clinical course of myocardial infarction in hospital phase: a case-control study. Cardiol. J. 20, 370–377. 10.5603/CJ.2013.0095 23913455

[B49] Zhang L. Desai N. R. Li J. Hu S. Wang Q. Li X.. (2015). National quality assessment of early clopidogrel therapy in Chinese patients with Acute Myocardial Infarction (AMI) in 2006 and 2011: insights from the China patient-centered evaluative assessment of cardiac events (PEACE)-retrospective AMI study. J. Am. Heart Assoc. 4:e001906. 10.1161/JAHA.115.001906 26163041 PMC4608074

