# Supplementary material for: Association between Myocardial Infarction and Periodontitis: A Meta-Analysis of Case-Control Studies
Source: Front Physiol. 2016 Nov 4;7:519. doi: 10.3389/fphys.2016.00519 (PMC5095113; doi:10.3389/fphys.2016.00519)
Supplement: Supplementary file 1 [file Table1.DOCX]

Supplementary Material

Association between Myocardial Infarction and Periodontitis: a Meta-Analysis of Case-Control Studies

Quan Shi ^1#^, Bin Zhang ^1#^, Na Huo ^1^, Chuan Cai ^1^, Hongchen Liu ^1*^, Juan Xu ^1*^

^1^ Institute of Stomatology, Chinese PLA General Hospital, Beijing, China

*** Correspondence:**Hongchen Liu
liuhc301@hotmail.com

Juan Xu
newxj@hotmail.com

^#^ These authors contributed equally to this work.

**Table S1. Summary of included studies**

| Study ID | Country | Type of control subjects | Dental examination | Main conclusion |
| --- | --- | --- | --- | --- |
| Rydén L 2016 | Sweden | Gender- and area-matched controls without MI | The periodontal condition was allocated to healthy, mild to moderate, severe periodontitis based on the bone height | The risk of a first MI was significantly increased in patients with periodontitis even after adjustment for confounding factors |
| Kodovazenitis G 2014 | Greece | Healthy subjects | Periodontitis was assessed using measurements of CAL, PD and number of missing teeth | The association between periodontitis and acute MI was consistent across different measurements and definitions of periodontitis |
| Willershausen I 2014 | Germany | Heathy controls matched by age, gender, number of residual teeth and smoking habits | A set of standard periodontal parameters, PD, BOP, and CAL, were recorded by clinical and radiological examination | Patients who have experienced a MI event had more missing teeth and a higher number of inflammatory processes, especially of endodontic origin, than healthy patients |
| Wożakowska KB 2013 | Poland | Patients with stable angina | The dental examination included dental history and physical dental examination with assessment of PI, CAL, PD, BI, and number of molar teeth | Poor oral health status, especially periodontal disease, may influence the occurrence and clinical course of MI |
| Khosravi Samani M 2013 | Iran | Patients without MI | All teeth excluding the third molar teeth were examined using the Michigan periodontal probe and the number of missing teeth was recorded | The results showed the presence of a significant association between periodontitis and MI that may serve as an indication of the importance of treating periodontitis carefully |
| Kodovazenitis G 2011 | Greece | Subjects without acute MI and with angiographically nonobstructive coronary disease | All subjects underwent an oral examination, and PD and CAL were used to measure periodontal condition | These findings implied that periodontitis may emerge as a novel target for reducing future risk in acute MI survivors |
| Holmlund A 2011 | Sweden | Age- and sex-matched controls from the same geographic area | A set of standard periodontal parameters, PD, BOP, and CAL, were recorded by clinical and radiological examination | Patients with MI had an impaired oral health compared to controls |
| Willershausen B 2009 | Germany | Controls were in good general health | All subjects were required to complete a questionnaire and underwent radiologic and oral examinations. | MI patients exhibited an less favorable state of dental health than did healthy patients, suggesting an association between chronic oral infections and MI |
| Stein JM 2009 | Germany | Controls without a history of CVD were matched by gender and age status | A comprehensive periodontal examination, including the assessment of PD and CAL, was performed by one dentist | This study confirmed an association between periodontitis and acute MI, finding that periodontal destruction was correlated with the presence of periodontal pathogens |
| Andriankaja OM 2007 | USA | Controls were identified from a random sample of residents of the same counties | CAL, PD, PI, and CI of the whole mouth were used to determine periodontal status | This study provided evidence of an association between PD and incident MI in both genders. This association appeared to be independent from the possible confounding effect of smoking |
| Kaisare S 2007 | India | Patients with coronary heart disease | All patients were clinically examined, including the assessment of PD, BOP, and missing teeth | The results of this study indicated an association between periodontal disease and acute MI |

| Andriankaja OM 2006 | USA | Controls were randomly selected from residents without MI | Periodontal disease was assessed using metrics including CAL, PD, and number of missing teeth | The association between periodontal disease and incident MI was consistent across different measurements and/or definitions of periodontal disease used. |
| --- | --- | --- | --- | --- |
| Cueto A 2005 | Spain | Trauma patients without MI | Recording of CAL and judgement of the degree of the periodontitis (absent, mild, moderate and severe) | There was evidence of an association between periodontitis and acute MI after adjusting for well-known risk factors |
| Renvert S 2004 | Sweden | Matched control subjects with no history of acute MI | Patients received a periodontal examination comprising dental radiographs and a comprehensive oral examination | The present study demonstrated using subject-based data that the proportion of BOP and plaque were significantly higher in subjects with acute MI |
| Deliargyris EN 2004 | Greece | Sex- and race-matched community volunteers without known heart disease | Each patient received a comprehensive periodontal examination, including measurement of PD and CAL | Periodontal disease was common in patients with acute MI and associated with an increased inflammatory response, as expressed by higher CRP levels |
| Rutger Persson G 2003 | Sweden | Matched control subjects with no evidence of CVD | A routine periodontal examination was performed by clinical and imaging examination | Patients at routine dental visits demonstrated evidence of bone loss around several teeth that was identified as a risk factor for future acute MI |
| Emingil G 2000 | Turkey | Patients with chronic coronary heart disease | All teeth, excluding third molars, were studied and clinical data, including missing teeth, PD, and BOP, were recorded | Periodontal disease may be associated with acute MI |

MI=myocardial infarction, CAL=clinical attachment loss, PD=probing depth, BOP=bleeding on probing, PI=plaque index, CI=Calculus index CVD=cardiovascular disease, CRP=C-reactive protein.
